# Psychedelic unselfing: self-transcendence and change of values in psychedelic experiences

**DOI:** 10.3389/fpsyg.2023.1104627

**Published:** 2023-06-14

**Authors:** Juuso Kähönen

**Affiliations:** Department of Philosophy, History, and Art, University of Helsinki, Helsinki, Finland

**Keywords:** values, self-transcendence, psychedelics, unselfing, egocentricity, moral epistemology, salience, self

## Abstract

Psychedelic experiences have been shown to both facilitate (re)connection to one’s values and change values, including enhancing aesthetic appreciation, promoting pro-environmental attitudes, and encouraging prosocial behavior. This article presents an empirically informed framework of philosophical psychology to understand how self-transcendence relates to psychedelic value changes. Most of the observed psychedelic value changes are toward the self-transcendent values of Schwartz’s value theory. As psychedelics also reliably cause various self-transcendent experiences (STEs), a parsimonious hypothesis is that STEs change values toward self-transcendent values. I argue that STEs indeed can lead to value changes, and discuss the morally relevant process of self-transcendence through Iris Murdoch’s concept of “unselfing”. I argue that overt egocentric concerns easily bias one’s valuations. Unselfing reduces egocentric attributions of salience and enhances non-egocentric attention to the world, widening one’s perspective and shifting evaluation toward self-transcendent modes. Values are inherently tied to various evaluative contexts, and unselfing can attune the individual to evaluative contexts and accompanying values beyond the self. Understood this way, psychedelics can provide temporarily enhanced access to self-transcendent values and function as sources of aspiration and value change. However, contextual factors can complicate whether STEs lead to long-term changes in values. The framework is supported by various research strands establishing empirical and conceptual connections between long-term differences in egocentricity, STEs, and self-transcendent values. Furthermore, the link between unselfing and value changes is supported by phenomenological and theoretical analysis of psychedelic experiences, as well as empirical findings on their long-term effects. This article furthers understanding of psychedelic value changes and contributes to discussions on whether value changes are justified, whether they result from cultural context, and whether psychedelics could function as tools of moral neuroenhancement.

Our states of consciousness differ in quality, our fantasies and reveries are not trivial and unimportant, they are profoundly connected with our energies and our ability to choose and act. If quality of consciousness matters, then anything which alters our consciousness in the direction of unselfishness, objectivity and realism is to be connected with virtue. (Murdoch, 2001, 84)

## 1. Introduction

This article aims to enrich our understanding of the value changes to which psychedelic experiences can lead. I argue that a significant reason for psychedelic value changes is self-transcendence—the reduction of egocentric ways of attributing salience and attention to the world around us—and the downstream effects. For example, in his autobiography, Albert Hofmann mentions meeting a young businessman:

He thanked me for the creation of LSD, which had given his life another direction. He had been 100 percent a businessman, with a purely materialistic world view. LSD had opened his eyes to the spiritual aspect of life. Now he possessed a sense for art, literature, and philosophy and was deeply concerned with religious and metaphysical questions. (Hofmann, 1980, 93)

This provides *prima facie* evidence that psychedelic experiences sometimes radically change one’s values. Not all value changes are radical: more commonly reported are moderate changes in various valuations and attitudes, or the ability to better (re)connect with pre-existing values (see Tables 1, 2).

**Table 1:** Definitions of central concepts.

Personal values (psychology)	Personal values refer to internalized cognitive structures that guide priorities and choices in life (Higgins, 2015; Oyserman, 2015)
Value (philosophy)	In philosophy, values are used to denote not only personal values, but also goods associated with various states of affairs, objects and contexts (i.e., beauty is a good associated with art, and justice as a good of the society). Philosophy is concerned with normative, epistemic and metaethical question concerning value, i.e., whether objects or state of affairs really are valuable, how this can be known and what values ontologically are (Tappolet and Rossi, 2015)
Salience	Salience refers to the importance (often automatically or subconsciously) attributed to various objects and aspects of experience, which makes certain objects or features stand out and be selected for attention (*cf.* Archer, 2022a,b)
Unselfing	Iris Murdoch coined concept ‘unselfing’ to refer to processes and experiences where salience attributed to self is reduced, and attention opens to the world and others (Murdoch, 2001)
Self-transcendence	The term ‘self-transcendence’ is used to refer both experiences and developmental processes of moving beyond one’s immediate self-boundaries and egocentric perspective, as well as developmental stages, motivation, personality traits, worldview and value orientations which emerges as a result of this process (Garcia-Romeu, 2010, Kitson, 2020)
Self-transcendent experiences	Self-transcendent experiences (STEs) refer to experiences where salience attributed to self is reduced and felt connection to the world and others is enhanced (Yaden et al., 2017). Self-transcendent experiences encompass experiences ranging from states of flow, to peak-experiences, awe and mystical experiences
Self-transcendent values	In Schwartz et al. (2012) value theory, self-transcendent values are a class of culturally universal values, in orthogonal relation to self-enhancement values. They consist of the subcategories of benevolence (valuations concerning strivings toward benefit of members of one’s ingroup) and universalism (strivings to benefit humanity, nature and other sentient beings in general).
Egocentricity (evaluative)	The degree to which salience and value are attributed to self, and the self is selected as the prominent context of evaluation, in contrast with other possible objects or evaluative contexts.

**Table 2:** Review of recent studies of values changes related to psychedelic use.

Value change	Is the change toward self-transcendent values?	Is the change conceptually associated with unselfing?	Studies	Type of study	Do authors link the changes to self-transcendent experiences?	How sustained changes were (i.e., when were last measurements conducted)?
Increased nature relatedness and/or increased appreciation of nature	Yes	Yes	Studerus et al. (2011)	Analysis of pooled data from eight double-blind studies in healthy volunteers	N/A	N/A
			Lyons and Carhart-Harris (2018)	Experimental study	In discussion connectedness is suggested as a possible mediating factor	Nature relatedness remained significantly increased 12 months post-dosing
			Kettner et al. (2019)	Prospective online survey	Changes were mediated by awe and ego-dissolutions	Nature relatedness was significantly increased 2 years after psychedelic experience
			Nour et al. (2017)	Survey	Ego-dissolutions mediated observed changes	N/A. Survey explored lifetime psychedelic use
			Forstmann and Sagioglou (2017)	Survey	Participants self-identification with nature predicted observed correlations, and authors suggest mediating effect of STEs	N/A. Survey explored lifetime psychedelic use
Increased pro-environmental behavior	Yes	Yes	Forstmann and Sagioglou (2017)	Survey	Participants self-identification with nature predicted observed correlations, and authors suggest mediating effect of STEs	N/A. Survey explored lifetime psychedelic use
Increased aesthetic appreciation of art and music	N/A (does not map to Schwartz theory)	Yes	Studerus et al. (2011)	Analysis of pooled data from eight double-blind studies in healthy volunteers	N/A	N/A
			Noorani et al. (2018)	Qualitative, interviews of therapy patients	Phenomenological reports suggest influence of STEs such as experiences of awe and interconnectedness	Interviews conducted 30 months after psychedelic experiences
			Masters and Houston (1966)	Qualitative, informal interviews and observational data from therapeutic practice	Phenomenological reports suggest influence of STEs	N/A
			Shanon (2002)	Qualitative, interviews of ayahuasca users and observational data	Phenomenological reports suggest influence of STEs	N/A
Increased altruism, prosocial behavior and/or concern for others	Yes	Yes	Griffiths et al. (2006)	Experimental study	Changes were mediated by mystical-type experiences	Changes were sustained 2 months after the experience
			Griffiths et al. (2008)	Experimental study	Changes were mediated by mystical-type experiences	Changes were sustained 14 months after the experience
			Griffiths et al. (2011)	Experimental study	Changes were mediated by mystical-type experiences	Changes were sustained 14 months after the experience
			Griffiths et al. (2018)	Experimental study	Changes were mediated by mystical-type experiences	Changes were sustained 6 months after the experience
			Noorani et al. (2018)	Qualitative, interviews of therapy patients	Phenomenological reports suggest influence of STEs such as experiences of awe and interconnectedness	Interviews conducted 30 months after psychedelic experiences
			Schmid and Liechti (2018)	Experimental study	Changes were correlated with mystical-type experiences	Altruistic/positive social effects and positive behavioral changes were significantly increased 12 months after the experience
Increased appreciation of spirituality	N/A (does not map to Schwartz theory)	Yes	Lerner and Lyvers (2006)	Survey	N/A	N/A. Survey explored lifetime psychedelic use
			Griffiths et al. (2018)	Experimental study	Changes were mediated by mystical-type experiences	Changes were sustained 6 months after the experience
Reduced valuation of financial success	Yes (by reducing self-enhancement value)	Yes	Lerner and Lyvers (2006)	Survey	N/A	N/A. Survey explored lifetime psychedelic use
More liberal and less authoritarian political views	No	No	Nour et al. (2017)	Survey	Ego-dissolutions mediated observed changes	N/A. Survey explored lifetime psychedelic use
Less authoritarian political views	No	No	Lyons and Carhart-Harris (2018)	Experimental study	In discussion connectedness is suggested as possible mediating factor	Authoritarianism remained decreased at trend level 12 month post-dosing
Reconnection to values	In some cases	In some cases	Belser et al. (2017)	Qualitative, interviews of therapy patients	Phenomenological reports suggest influence of STEs	N/A
Decreased specisism and increased animal solidarity	Yes	Yes	Pöllänen et al. (2022)	Survey	Ego-dissolutions were associated with decreased specisism, increased animal solidarity and increased desire to help animals	N/A. Survey explored lifetime psychedelic use

The table above reviews recent studies of value changes related to psychedelic use the author has been able to find (note that the same study can be listed in multiple categories). The table maps whether the value changes reported in the studies are changes toward (a) self-transcendent values (according to Schwartz et al., 2012, classification) and (b) conceptually associated with unselfing (defined as reduced salience attributed to self and increased attention and concern to the world and others). The table above also documents (1) the type of the studies, (2) whether STEs correlated with or mediated the value changes or if they were discussed by authors as potential causes, and (3) information regarding how sustained changes were.

No research has explicitly attempted to theoretically integrate and explain the value changes to which psychedelics can lead. To embark toward such integration, I review in Section 2, the literature on psychedelic value changes and argue that many such changes are changes toward self-transcendent values and are associated with various self-transcendent experiences (STEs).

In Section 3, I combine philosophical argumentation and empirical evidence to develop an explanatory framework for value changes in relation to a form of self-transcendence I call “unselfing” (Murdoch, 1997, 369). This framework gives a plausible explanation for why STEs might lead to self-transcendent values. My key philosophical claim is that selfhood and salience attributed to self are one central factor modulating how individuals intuit and grasp values. The central hypothesis is that overt egocentricity can easily bias valuations, reducing the importance of self-transcendent values. I claim that freeing of attention and salience from egocentric concerns enables an opportunity for perspectival and evaluative changes—opening our attention and concern to wider contexts, values, and frames of reference. Theorists in various disciplines converge on similar ideas, ranging from the philosophical psychology of Iris Murdoch, through the late developmental theories of Maslow and Kohlberg, to recent psychological work on “the quiet ego” and “hypo-egoic phenomena” (Leary and Guadagno, 2011; Wayment and Bauer, 2017).

In Section 4, I discuss psychedelic value changes in light of this framework and link them to psychological research and theoretical understanding of psychedelic experiences. Arguably, by reducing egocentric evaluative biases and increasing the attribution of attention and value outside the self, unselfing psychedelic experiences can connect and align us to the world and provide better epistemic access to self-transcendent viewpoints and values.

In Section 5, I discuss the implication of the framework. The proposed framework contributes to moral and epistemological discussions of psychedelics. Positing unselfing as a source of value change gives initial moral-epistemic justification for self-transcendent value changes (Lavazza, 2017; Langlitz et al., 2021), potentially legitimizing psychedelics as a societally valuable source of moral enhancement—a pharmacological means to improve humans’ moral state (Earp et al., 2017; Earp, 2018).

An important caveat of this article is that self-transcendent value changes occur only in a subset of psychedelic experiences, presumably more likely in certain contexts or use patterns and conditioned by the pivotal influence of set and setting (i.e., personal and contextual factors), discussed further in Sections 3.9 and 4.4. Furthermore, self-transcendence is not the only factor contributing to psychedelic value changes (Pace and Devenot, 2021). Charting all factors and mechanisms causing and modulating these changes, as well as the various types of value changes and their relation to self-transcendence, is an important future task. It should also be noted that this article uses “mechanism” in the general sense of “a natural or established process by which something takes place or is brought about” (Oxford English Dictionary), rather than as a strictly biological or neural concept (*cf.* van Elk and Yaden, 2022).

## 2. Review of psychedelic value changes and self-transcendent values

The concept of self-transcendent values is from Schwartz et al.’s (2012) value theory,^1^ which classifies certain culturally universal values as self-transcendent. In this article, I loosely follow this usage, although self-transcendent values could be more broadly defined as personal values oriented beyond individual’s gains and losses, implying an attribution of value outside the self for non-egocentric reasons. This broader definition of self-transcendent values includes the valuations associated with esthetics and spirituality.

As detailed in Table 2, recent empirical literature links psychedelics to long-term increases in:

- Appreciation of nature (Studerus et al., 2011), non-human animals (Pöllänen et al., 2022), nature relatedness (Nour et al., 2017; Lyons and Carhart-Harris, 2018; Kettner et al., 2019), and pro-environmental behavior (Forstmann and Sagioglou, 2017);
- Esthetic appreciation (Studerus et al., 2011; Noorani et al., 2018);
- Altruism and prosociality (Griffiths et al., 2006, 2008, 2018; Schmid and Liechti, 2018);
- Appreciation of spirituality (Griffiths et al., 2018);
- Reduced valuation of financial success (Lerner and Lyvers, 2006);
- More liberal and less authoritarian political views (Nour et al., 2017; Lyons and Carhart-Harris, 2018).

The link between self-transcendent values and psychedelics has not been proposed in the research literature, although it is suggested in Peck’s (2020) recent popular article.^2^ Overall, the above body of research supports the claim that psychedelic experiences can change values, valuations, and life priorities toward self-transcendent values: all of these studies except those concerning political orientation evidence change toward self-transcendent values.

Notably, these psychedelic changes of values often occur via the mediation of STEs. The connection between a particular value change and STEs (such as ego-dissolution, awe, or mystical experiences) is noted in many of these studies. Still, theoretical integration is lacking. To fill this research gap, I make the hypothesis that STEs and the long-term changes they induce provide a more general explanation for psychedelic value changes. Already, the same self-reducing mechanisms are considered central to the therapeutic and existential effects of psychedelics (Letheby, 2021, 197, 202; Letheby, 2022; van Elk and Yaden, 2022). Furthermore, there are plausible links among STEs, changes in many psychological variables induced by psychedelics, and self-transcendent values, explored in Section 4. The next section aims to philosophically articulate a way to understand this connection between self-transcendence and values.

## 3. Self, unselfing, and value change

### 3.1. Values and salience

There are various views on and approaches to values (e.g., Rokeach, 1973; Brosch and Sander, 2015; Oyserman, 2015; Hobbs, 2021). From a psychological viewpoint, values are abstract beliefs or cognitive structures that govern priorities and affective reactions and guide goals and choices (Oyserman, 2015; Schwartz, 2015). Values are part of the overall process of how a person situates and orients himself in the world, together with higher-order aspects of cognition such as worldview (Koltko-Rivera, 2004) and lower-level evaluations such as experiential valence, salience, and affective processes (Archer, 2022a,b; Varela et al., 1993).

In contrast to values, salience can be defined as what is experienced as important in the present moment (Watzl, 2022; Whiteley, 2022; Archer, 2022a,b). In our immediate experience, we find certain things or properties important while ignoring others. What is salient is a complex result of various contextual factors, unconscious processes, and the deliberate use of attention; it also changes spontaneously and fluidly between different moments and framings.

There are multiple connections between values and salience. Attribution of value occurs to a large extent as non-inferential and automatic attribution of salience guiding action and perception. Also, what is salient at any given moment modulates which values are activated. Conversely, our abstract values modulate how we attribute salience and attention. For example, Archer (2022a,b) argues that salience should be conceived in a complex dynamic relation to our evaluative worldview—various abstract “standing judgments” and values that affect salience through their context-dependent application and realization in particular situations.

### 3.2. The evaluative self

The notion of “self” is notoriously complex to define, causing much conceptual and theoretical confusion (Leary and Tangney, 2012). Here, I focus on an ordinary, basic sense of self: identification with and/or sense of ownership of a body and personhood, leading to perceiving oneself as a distinct and enduring entity separate from other entities in the world (Albahari, 2006).

A crucial evaluative function the self enables is the filtering of sensory data according to its importance. The machinery of selfhood in many ways constricts and dictates what is tagged as salient and worthy of attention (Letheby and Gerrans, 2017, 4). Cognitively and neurally, self-relevance influences many disparate processes: we pay more attention to stimuli that are self-related (Millière, 2017, 10–13; Sui and Humphreys, 2015). The world is a complex, chaotic place, and humans must narrow their perception to patterns relevant to survival. Constructing a self, or *selfing,* is intimately related to these utilitarian, evolutionary pressures to seek and avoid various things in the world (Huxley, 1954; Letheby and Gerrans, 2017).

I use egocentric salience to describe salience filtered through egocentric evaluative biases, resulting from identification and investment in personal self and its goals (Albahari, 2006, 60). This whole machinery of selfhood and associated egocentric salience creates an egocentric frame of reference into our experience, guiding the direction of attention and what features are considered salient (Letheby and Gerrans, 2017, 4).

Egocentric salience is related to both narrative and embodied aspects of self, and functions as a bridge between these aspects of selfhood (Letheby and Gerrans, 2017, 3; Millière et al., 2018). Narrative self adds whole layers of identity on top of the embodied self, affording the ability via thought and imagination to fix attention to past or future events relevant to the self and symbolically extend self (for example, by identifying with one’s possessions and opinions).

Noting the possibility to conflate various meanings of egocentric used in the literature, I refer here exclusively to attributions of salience.^3^ Similarly, egocentricity and egocentric salience in the present context should not be conflated with egoism: egocentric salience does not imply that one is egoistic in morally problematic ways. I reserve the term egoistic for extreme, morally problematic forms of egocentricity such as narcissism and the inability to care for others, assuming that most of our everyday experiences are egocentric to a morally acceptable degree. Another potential confusion is that, in some sense, all personal goals are egocentric as they are tied to self: for example, Wiese (2019) explores the idea that selfhood is constructed through the integration of salience attributed to various goals. Ego-structures can, adaptively and functionally, assimilate and serve self-transcendent goals and values—ego optimally facilitates our connection to the world. I mostly use egocentric in a narrower sense: denoting concerns serving and related to oneself, associated with mundane concerns with oneself, and the self-enhancement values (Schwartz, 2012).

### 3.3. Overt egocentricity as a falsifying veil

The capacity for egocentric attribution of salience is hugely adaptive—egocentric pursuits are a necessary part of human life. Still, for both existential and moral reasons, the degree of egocentricity matters. Humans are prone to situations where the salience attributed to self grows disproportionately strong, occluding wider concerns and values from our perspective. The philosopher Iris Murdoch beautifully thematizes how egocentric tendencies can limit our perspective:

By opening our eyes we do not necessarily see what confronts us. We are anxiety-ridden animals. Our minds are continually active, fabricating an anxious, usually self-preoccupied, often falsifying veil which partially conceals our world. (Murdoch, 2001, 84)

I interpret that this “falsifying veil” denotes a cognition in which excessive value and focus are placed on egocentric ways of perceiving. Instead of enabling our connection to the world, the self sometimes works like an over-active filter, blocking from our vision everything other than egocentric pursuits. Murdoch referred to strongly egocentric forms of cognition as “fantasy,” entailing “the proliferation of blinding self-centered aims and images” that reduce everything to the false unity of self (Murdoch, 1997, 354). Murdoch regarded this tendency to value only things connected to our concerns and with utility for the self as a central obstacle to directing our attention and imagination to the world (Denham, 2001, 624). In this sense, Murdoch claimed that the self is a place of illusion (2001, 93), entailing a possibility of a vicious self-reinforcing feedback loop between egocentric salience and sense of reified selfhood.

Murdoch is not alone in contending that egocentric motivations are often so strong as to limit our interests, scope of caring, and motivations for non-egocentric pursuits (Murdoch, 2001, 99). For example, Maslow (1971, 249–255), Fromm (1976), Schopenhauer (1818; see Denham, 2001, 623–624), and Buddhist traditions (Albahari, 2006, 24–27, 164; Burbea, 2014) all recognize this link between overt egocentricity and certain existentially and morally limiting modes of orienting, acting, and being in the world.

There are many common situations in which overt egocentricity occurs. States of suffering and stress tend to make people egocentric, while overt egocentricity can itself cause suffering, creating a feedback loop reinforcing disconnection to non-egocentric values (Leary and Guadagno, 2011, 139–40; Vervaeke et al., 2017). Karen Horney (1999 [1950]) suggested that neuroses always make one egocentric. For example, extreme rumination and associated inertia can make one self-centered to a dysfunctional degree. Similarly, addictive desires might divert extensive motivation away from other aims of greater importance (Vervaeke and Ferraro, 2013). Even highly functioning individuals can be drawn into the overtly egocentric pursuit of pleasure, fame, wealth, and power; extremely so in personality features dubbed “the dark triad” (Furnham et al., 2011).

### 3.4. Unselfing

Given these relatively strong forms of egocentric tendencies, it is easy to understand why unraveling the overt attribution of self-saliency is beneficial. I adopt Murdoch’s term “unselfing” to stress both the existential and moral importance of reducing egocentric salience. Unselfing points to moments and processes where our attention opens and we break our psychological isolation from the world around us (Murdoch, 1992, 17; Driver, 2020, 169–170). Considering previous sections, we can interpret unselfing as a reduction in egocentric salience and a concomitant increase in our capacity and willingness to become concerned with what is non-self. For example:

The most obvious thing in our surroundings which is an occasion for ‘unselfing’ is what is popularly called beauty […] I am looking out of my window in an anxious and resentful state of mind, oblivious of my surroundings, brooding perhaps on some damage done to my prestige. Then suddenly I observe a hovering kestrel. In a moment everything is altered. The brooding self with its hurt vanity has disappeared. There is nothing now but kestrel. And when I return to thinking of the other matter it seems less important. (Murdoch, 2001, 84)

The beauty of the kestrel is a value perceived in the outside world and occasions a shift to a less egocentric mode of attention. Unselfing can occur in various ways, encompassing appreciation of nature, art, contemplative practices, and intellectual disciplines—basically anything that involves non-egocentric attention to reality and recognition of value(s) lying outside the self (Murdoch, 2001, 84–86, 90–91; Denham, 2001, 624).

For Murdoch, at the core of unselfing is loving attention, a term she borrows from Simone Weil and describes as “a just and loving gaze directed upon an individual reality” (1997, 327). She elaborates thus:

It is in the capacity to love, that is to see, that the liberation of the soul from fantasy consists. […] What I have called fantasy […] is itself a powerful system of energy […] What counteracts the system is attention to reality inspired by, consisting of, love. (Murdoch, 1997, 354)

Murdoch regards loving attention (and associated non-egocentric imagination) as a movement outward from the self, enabling progress toward a more accurate perception of the world (Panizza, 2019, 3–4, 15; Chappell, 2018; Driver, 2020). Loving attention—especially when combined with honest self-criticism—counteracts our egocentric fantasies and allows for overcoming our biased perspectives and those “convincingly coherent but false pictures of the world” that we so easily build (Murdoch, 1997, 329; see also Driver, 2020, 174–175).

For Murdoch, progress and virtue necessarily require the ability to see through egocentric structures of valuation, as this is the only way to truly understand what goodness is (Murdoch, 2001, 103; Mole, 2022). Loving (i.e., disinterested and non-egocentric) attention leads to moral progress by changing our perspective, which is the root of our reasoning and actions (Olsson, 2018, 168–169; Driver, 2020, 173–174). Only by opening our attention to the surrounding world, can we transcend our limited views, conceptions, and sensibilities, and learn to appreciate and respect that which is not ourselves, for example, by understanding an artistic work or another person more deeply. Through self-correction and learning enabled by the use of loving (or non-egocentric), one can refine one’s evaluations and moral concepts and conceptions, which largely dictate how one concretely understands good and other abstract values (Murdoch, 1992, 325; Murdoch, 1997, 307, 313, 317–323). Wright (2005) denotes this process as “sensibility transcendence.” Thus, transcending the falsifying veil of self-absorbed fantasy is valuable for both moral and epistemic reasons (Denham, 2001; Murdoch, 2001, 84, 93, 97; Panizza, 2019).

### 3.5. Converging constructs

In psychological research, phenomena similar to unselfing have been approached through various constructs such as “hypo-egoic states” (Leary and Guadagno, 2011), “self-transcendent experiences” (Yaden et al., 2017), and “peak-experiences” (Maslow, 1970, 1971). All these are umbrella concepts denoting a variety of experiences that share a common core of (1) reduced self-awareness (or reflection about oneself), (2) reduced ego-involvement and egocentric salience, and (3) altered self-boundaries and sense of increased connection or interconnectedness to the world. I suggest that the phenomena discussed under these concepts almost always involve unselfing, i.e., directing attention and evaluation away from the self and egocentric concerns.

These constructs converge on the idea that our states vary according to the level of egocentricity, and that loosening egocentricity leads to shifts in our perspective on or position in relation to the world. In these experiences, the center of evaluation called “self” dissolves to various degrees, and the way we evaluate and pay attention alters. Becoming less entangled in and fixated by our egocentric biases, concerns, and wants, we can see the world from a wider perspective and our attention is more available and disinterested (Maslow, 1970, 78; Albahari, 2006, 51–74). In these kinds of states, the self might be conceived as more interconnected and relational and be more permeable to the world and others (Piff et al., 2015; Hanley et al., 2017; Yaden et al., 2017).

### 3.6. Unselfing and value change

I claim that these perspectival changes associated with unselfing can lead to changes in values. In general, values are perspectival. Which of our values are “active”—i.e., guiding our behavior and goal-framing—in any given moment is context-dependent, influenced by various conditions and factors (Schwartz, 2012; Schwartz et al., 2012; Oyserman, 2015). One key factor is what appears salient in the individual’s conscious experience at any given moment. In turn, what is salient depends largely on the given context of evaluation. For example, an evaluation centered on one’s economic prosperity will arouse different values compared to one focused on the survival of humanity. Conversely, possessing certain values tends to make certain contexts of evaluation more salient: for instance, if I value nature, I tend to ponder economic activities and my personal choices from the viewpoint of their impacts on nature.

In this sense, our personal values emphasize and make salient certain contexts of evaluation and associated goods. The given evaluative context and values one grasps can be conceived as mutually arising aspects of the same evaluative activity. In this sense, values are tendencies to evaluate from particular perspectives—seeing certain evaluative contexts as salient enough to trump consideration of other contexts.

I propose that attributions of egocentric salience foreground the **self as an evaluative context**. When egocentric concerns are sufficiently salient, the associated egocentric framings and attributions of salience filter which values or goods are intuited or grasped, nudging evaluation toward self-enhancement. The salience biases created by egocentric fantasies and goals thus instigate a structure of values that easily disconnects us from non-egocentric values. This line of thinking is also supported by and could be used to explain a central tenet of Schwartz et al.’s (2012) theory: the orthogonal relation between self-transcendent and self-enhancement values. Specifically, egocentric biases are either on or off, modulating whether our values center around the self or self-transcendent evaluative contexts.

I hypothesize that reducing egocentric salience and attention can lead to both momentary shifts and long-term changes in personal values by allowing a transition to a wider perspective, opening a person to values (or goods) beyond egocentric goals and considerations. Unselfing can be pictured as a continuum of progressively less egocentric ways of framing the relation between ourselves and our environment. Deconstruction of egocentric attributions of salience and attention allows contextualizing of the self to wider frames of reference and associated expansion in evaluative perspective and values. These perspectival changes are reflected in various modalities such as the cognitive processes of thinking and imagination, in our perceptual or experiential salience, and in our behavior.

I argue that this perspectival widening through unselfing drives a shift toward relatively more intrinsic and self-transcendent modes of attributing value, thus helping one to grasp non-egocentric values. Unselfing can allow for value-instigating experiences and perspectives, or activate existing self-transcendent values. Consequently, experiential salience might become more tuned with the values connected to goods of wider spatial, temporal, or social contexts, thereby expanding our scope of care and concern.

Perspective can open to the **immediate present-moment context** surrounding us and to its non-instrumental value(s). When one is more present, the narrative structures of selfhood required for instrumental goals operate less strongly. When less entangled in our plans and past and future, and less anxious about our gains and losses, we can more readily perceive the intrinsic value of particulars, such as the value of objects of nature and art. Unselfing might thus lead to “non-egocentric respect for the particular” (Nagel, 1986, 222–223).

Furthermore, perspective can open to **allocentric contexts—**interpersonal and relational contexts spanning from consideration of loved ones and an ingroup to universal concern for other persons and humankind. Benevolence values in Schwartz et al.’s (2012) classification and prosocial values, in general, are associated with the recognition of these contexts as salient.

Finally, perspective can open to vast contexts setting the self as part of still wider frames of reference, such as nature or the cosmos at large. These could be termed **cosmocentric contexts** (*cf.* Maslow, 1970, 96). The idea that universal values are maximally accessible from a minimally egocentric perspective is already found in philosophy of Plato’s (2008). Murdoch (2001, 101–103)—who was heavily influenced by Plato—claimed that goodness is connected with an attempt to see the unself, i.e., the movement toward a non-egocentric perspective. She elaborated thus:

Goodness is connected with the acceptance of real death and real chance and real transience and only against the background of this acceptance, which is psychologically so difficult, can we understand the full extent of what virtue is like. The acceptance of death is an acceptance of our own nothingness which is an automatic spur to our concern with what is not ourselves. (Murdoch, 2001, 103)

Seeing clearly the vastness of the world and the ephemerality of our self very understandably alters valuations. The same thrust to acknowledge our mortality, see through our self-centered perspective, and marshal a shift in values and ways of living has arguably long motivated religious and spiritual traditions (Hadot, 1995, 2004; Leary and Guadagno, 2011, 143; Albahari, 2014; Pelser and Roberts, 2015; Thompson, 2020).

In Murdoch’s thinking, unselfing is associated with the capacity to put things in the right perspective by recognizing various values in the world (Driver, 2020, 171). In this line of thinking, self-transcendence and unselfing could be understood as processes of honing our conceptions of what should and should not matter. Opening attention to various evaluative contexts and associated goods is a necessary antecedent for putting things in the right perspective and refining one’s values. As Murdoch claimed, letting go of egocentrism occurs naturally when we grasp non-egocentric values (Driver, 2020). Unselfing might thus reduce the discrepancy between what we see as important given our egocentric biases and what is more important in a wider context, and enable one to form more encompassing values than self-enhancement values.

### 3.7. Support for the framework

Both empirical research and theoretical models support the notions that (1) there are substantial long-term and trait differences in egocentricity, (2) self-transcendent developmental stages are associated with self-transcendent values, and (3) momentary experiences of reduced egocentricity can contribute to self-transcendent values.

#### 3.7.1. Trait differences in egocentricity

Various psychological constructs describe trait-level differences in egocentricity. Wayment and Bauer (2017) use “quiet ego” (contrasted with “noisy ego”) to describe traits associated with compassionate self-identity and the ability to transcend egoistic concerns and adaptively balance the needs of others and self. Dambrun and Ricard (2011) draw a similar trait-level distinction between selflessness and self-centeredness. Maslow (1971, 241–255) associates self-transcendence with non-egocentric “being-cognition” and its corollary “being-motivation,” and contrasted these with “deficiency-cognition” and “deficiency-motivation,” involving orienting oneself to the world through unfulfilled egocentric needs. Fromm (1976) contrasts two fundamental evaluative orientations or existential modes: the non-egocentric “being mode” and the egocentric, goal-oriented, and alienated “having mode.”

Many theories converge on the notion that expanding one’s perspective to universal concerns is essential for achieving mature development of human cognition and morals (Loevinger and Blasi, 1976; Kegan, 1982; Gibbs, 2003), or explicitly link self-transcendence to these higher developmental stages (Maslow, 1971; Kohlberg and Ryncarz, 1990; Levenson et al., 2001). Similarly, the expansion of self–other boundaries is theoretically central to many notions of developmental self-transcendence (St. Arnaud, 2019). Researchers have linked wisdom with the ability to transcend one’s narrow perspectives and self-interests and with the development of a non-egocentric perspective on life (Le and Levenson, 2005; Ardelt, 2008; Vervaeke and Ferraro, 2013; Aldwin et al., 2019).

#### 3.7.2. Trait or developmental self-transcendence and self-transcendent values

Multiple authors explicitly connect trait differences in egocentricity to self-transcendent values. Wayment and Bauer (2017, 83) identified strong empirical correlations between quiet ego and (a) prosocial concerns, (b) compassionate goals, and (c) self-transcendent values of Schwartz’s classification. Researchers have also developed measurements for Fromm’s existential modes and observed a conceptual similarity between these and the value orientations of Schwartz’s value theory (Cohen et al., 2005).

Furthermore, there are theoretical frameworks linking developmental self-transcendence to both STEs and value change. Maslow suggested that peak-experiences can lead to temporary states of “being-cognition,” in which the world is perceived through a set of values and qualities he called “being-values” or “intrinsic values of Being” (B-values), such as the Platonic triad of goodness, beauty, and truth (Maslow, 1970, 64–65, 96; Maslow, 1971, 186, 286–328). For Maslow, B-values were biologically rooted, transculturally shared, and “cosmocentric”—not based on social or egoic concerns. A shift in perception to these B-values forms another kind of gestalt to organize the perceived world, “a change in attitude, valuing reality in a different way, seeing things from a new perspective, from a different centering point” (Maslow, 1970, 78). B-values describe reality and imply a normative aspect, giving direction on what should be pursued; their introjection into one’s value structure results in non-egocentric “metamotivation” (Maslow, 1971, 286–328).

Similarly, in later formulations of moral development theory, Kohlberg and Ryncarz (1990) speculated about a (metaphorical) seventh “cosmic” stage of moral development, beyond the sixth stage of universalizable ethical thinking (Gibbs, 2003, 70–72). They saw that the existential crisis from confronting the “finitude of our individual self” could cause a gestalt shift in self-understanding, leading one to identify “with the cosmic or infinite perspective and value life from its standpoint” (Kohlberg and Ryncarz, 1990, 192–196). Kohlberg and Ryncarz (1990, 200–201) speculated that this shift occurs through a radical transcendence or decentering of the egocentric viewpoint, with significant normative implications such as finding harmony with and love of the cosmic order, and understanding that power and pleasure are not intrinsic ends of human life (Gibbs, 2003, 70–72). Converging with Maslow, Kohlberg, and Ryncarz (1990, 192, 206) saw that mystical experiences or “experience of nonegoistic or nondual variety” might be required for this kind of gestalt shift in moral understanding and valuations.

#### 3.7.3. Self-transcendent experiences and self-transcendent values

Evidence from non-psychedelic research supports the association of STEs with self-transcendent values. Awe has been observed to cause increases in prosocial behavior and generosity (Piff et al., 2015). Near-death experiences, which often involve ego-dissolution, have been observed to result in less materialistic values and increased connection to nature (Greyson, 1983; Gandy, 2017; Barberia et al, 2018; Martial et al., 2021; Sweeney et al., 2022). Nature immersion has been observed to lead to not only pro-environmental behavior but also generosity, prosocial values, and more intrinsic values in general (Weinstein et al., 2009; Zhang et al., 2014; Lumber et al., 2017; Kettner et al., 2019). Isham et al. (2022) argue that STEs can promote ecological wellbeing, a notion intrinsically associated with considering non-egocentric matters such as other beings and nature, and review evidence for STEs possessing a general tendency to enhance pro-environmental and self-transcendent values.

### 3.8. Normative issues

The argument above might evoke normative questions on what degree of egocentricity an individual should have. According to this argument, value changes associated with unselfing are normatively desirable: personal values should be informed by evaluative contexts surpassing the good of the individual ego. Various thinkers and traditions converge on the view that developing a wider or more encompassing perspective beyond narrow self-interest and aligning and connecting with goods beyond the self is both a moral requirement and integral to human development (Maslow, 1970, 1971; Nagel, 1986; Kohlberg and Ryncarz, 1990; Murdoch, 2001). If we admit that genuine improvement in one’s evaluations results from self-transcendence, self-transcendence has to be normatively desirable.

Admittedly, the question of how much self-transcendence is required for human flourishing or morality is complex. The exact standards for the normatively required level of egocentricity depend on many substantial value-and worldview-laden questions such as metaethical stances, which cannot be fully explored in this article.^4^ I certainly do not argue that we should always be in states of utter self-transcendence—egocentric values have a place in human life. Too much self-transcendence or exclusive “view from nowhere” might even be morally problematic as it completely abstracts away particular subjective viewpoints and concerns (Nagel, 1986). Relatively uncontroversially, I advocate unselfing to the extent required for balancing our egocentric interests with consideration of wider goods: this is normatively desirable and helps to refine one’s values and conceptions of the good (Murdoch, 1992, 325; Murdoch, 1997, 307, 313, 317–323; Wright 2005; *cf.* Leary and Guadagno, 2011, 143–144).

### 3.9. Contextual factors and issues of long-term changes

The framework presented above supports the notion that unselfing can plausibly change individuals’ values. Since the pioneering work of William James, the occurrence of rapid and sustained change in one’s outlook, worldview, beliefs, and values from momentary experiences is well attested and discussed under various terms such as “quantum change” and “pivotal mental experiences” (Miller and C’de Baca, 2001; Koltko-Rivera, 2004; James, 2008; Brouwer and Carhart-Harris, 2021; Timmermann et al., 2021; Yaden and Newberg, 2022).

Notably, STEs do not always lead to value changes. There are three stages in the process of value change that I have described above: (1) reduction of egocentricity; (2) tuning into non-egocentric goods and contexts of evaluation, and (3) long-term changes as these perceived goods are introjected into one’s motivational structure. It is likely that each of these steps creates a window of opportunity for the next one to take place, but does not necessarily lead to the next stage.

There are currently many unresolved questions concerning how and when momentary experiences lead to long-lasting changes, and which overall conditions and determinants are required. Multiple factors possibly affect the translation of STEs into self-transcendent values. Examples include:

1. Psychological conditions and actions of the individual, such as intention and desire for self-transcendence, a sufficient developmental stage, personality factors, and efforts to prepare, integrate, and reflect on experiences (St. Arnaud and Sharpe, 2022);
2. Prior predisposing values and worldview, enabling one to see self-transcendence and self-transcendent values as meaningful goals or aspirations, thus fostering the ability to interpret experiences in ways that support value change (Koltko-Rivera, 2004, 17; St. Arnaud, 2019);
3. Availability of viable cultural frameworks for interpreting these experiences (*cf.* Koltko-Rivera, 2004, 14). Value transmission is heavily influenced by culture (Boer and Boehnke, 2015), and the effects of singular experiences cannot be disentangled from the wider cultural matrix of values in which the individual is embedded. How experiences and their implications are interpreted depends on a person’s cultural surroundings and worldview, particularly because experiences are plausibly affected or even constructed in interaction with the cultural context and meaning-making systems (Katz, 1978; Hartogsohn, 2020; Dupuis, 2021; Dupuis and Veissiere, 2022);
4. Engagement in practices and social contexts that support long-term cultivation of self-transcendent values after singular pivotal experiences. Historically, STEs have often been embedded in wider cultural traditions providing a matrix of supporting social conditions and an ecology of practices to facilitate change (Hadot, 1995; Hunt, 2013; Yaden et al., 2020).

In summary, deconstruction of evaluative machinery around the self, or even temporary tuning into wider evaluative contexts might not always be enough for sustained change in values. As many developmental theories recognize, value formation is a complex process. A full theoretical account of value change through STEs should consider relevant contextual factors such as the individual’s developmental stage, the role of cultural context, and engagement in the long-term cultivation of self-transcendence.

## 4. Psychedelic unselfing and change of values

Letheby (2017, 2021, 196–204) claims that psychedelics’ ability to induce unselfing is core to psychedelic therapy and spirituality. Unselfing for Letheby denotes the deconstruction of the self-model (constructed sense of self) and the consequent liberation of attention from its constraints—breaking down the narrow walls of ego. As Letheby (2021, 220) explains:

When phenomenal reality is filtered and structured less strongly through the goals and preferences of a reified, essentialised self, we can experience wonder, awe, broader perspectives, and feelings of profound kinship with the entirety of manifest existence.

Similarly, I suggest that psychedelics’ ability to transform values and sensibilities is closely tied to reducing egocentricity and occasioning self-transcendent perspectives. I propose that by deconstructing egocentric salience psychedelic experiences can open our attention to the world and widen our evaluative context to encompass the immediate environment, the whole context of our personal life, our ingroup, and even broader social, ecological, and cosmological contexts (*cf.* Whitfield, 2021). By tuning into these non-egocentric contexts and evaluative modes, one gains better epistemic access to various self-transcendent values, which sometimes leave lasting imprints on personal values.

Next, I chart the changes toward self-transcendent values associated with psychedelic experiences, using categories from Schwartz et al.’s (2012, 669) value theory, supplemented by reconnection to values, esthetic and spiritual values, and then explore related theoretical issues.

### 4.1. Self-transcendent psychedelic value changes

#### 4.1.1. Reconnection to values

In Belser et al.’s (2017) study, all 13 cancer patients who suffered from anxiety described “revised life priorities” as a major effect of psilocybin therapy:

These participants came to **“remember” during their psilocybin session what to them was most important about life.** […] “We forget what’s really important; we get carried away with work and making our money and paying our bills, and this is just not what life is about.” Participants **were compelled to reorient their lives afterward** in a way that continued to connect them to a similar place. (p. 374, emphasis added)

These participants described shifts in their life priorities away from instrumental pursuits toward more fundamental objects of valuation, similar to the observation of reduced valuation of economic success by Lerner and Lyvers (2006). Swift et al. (2017, 24) observe that these same participants were pulled away from habitual patterns and overwhelm caused by cancer and “given an expanded perspective on what was felt to be most important and meaningful in life, which endured beyond the session,” allowing patients to reconnect to life, their authentic selves, and the wider world beyond their sickness. As argued in Section 3.3, suffering tends to narrow the evaluative perspective—a sense of disconnection is associated with mental health issues (Carhart-Harris et al., 2018a)—but psychedelic experiences can reverse this trend, as illustrated by the case of a terminal cancer patient:

It was less about my illness. I was able to put it into perspective. […] Not to see oneself with one’s sickness as center. There are more important things in life. […] The evolution of human kind for example. […] Your Inner Ego gets diminished, I believe, and you are looking at the whole. (Gasser et al., 2015, 62)

These changes might plausibly be explained by a widening of the evaluative context to the full context of one’s life and beyond as a result of reduced egocentric salience. As egocentric pursuits and worries likely become relatively less important, there is space for attunement with pre-existing core values from which one was disconnected—which often are self-transcendent values.

#### 4.1.2. Esthetic values

Beauty is perhaps the most commonly grasped intrinsic value in psychedelic experiences. Examples abound of profound esthetic psychedelic experiences (Huxley, 1954, 4–6; Masters and Houston, 1966, 156–165; Shanon, 2002, 176). Such experiences sometimes lead to a sustained appreciation for art and the beauty of nature (Vaughan, 1983; Shanon, 2002, 176; Studerus et al., 2011; Noorani et al., 2018). Exploring the phenomenology of ayahuasca experiences, Shanon (2002, 176) claims that these experiences heighten the esthetic perception to the extent that “the ayahuasca experience is cardinally aesthetic.” This fits with the above framework, as unselfing frees up attention and salience, allowing deeper attunement to the present sensory environment.

#### 4.1.3. Benevolence values

Psychedelic unselfing can foster allocentric perspectival widening, opening our concern and care to interpersonal relations and social contexts in which we are embedded and leading to a better grasp of relational values and the intrinsic value of other persons. Experiences of relational embeddedness, social connectedness, identity fusion, and other relational processes are common in psychedelic experiences (Belser et al., 2017; Kettner et al., 2021; Newson et al., 2021; Roseman et al., 2021; Weiss et al., 2021). Moreover, experimental studies have observed increased prosociality and altruism (Griffiths et al., 2008, 2011, 2018; Noorani et al., 2018; Schmid and Liechti, 2018). These prosocial changes are likely associated with feelings of empathy and sentiments of love and compassion, all common self-transcendent emotions in psychedelic experiences (Shanon, 2002, 157, 164, 339; Pokorny et al., 2017; Watts et al., 2017, 535; Blatchford et al., 2020; Mulukom et al., 2020; Letheby, 2021, 202). These changes fit the category of “benevolence” in Schwartz’s self-transcendent values, defined as devotion to ingroup members’ welfare (although many of these changes overlap with more universal concerns).

#### 4.1.4. Nature values

Psychedelic experiences can foster nature relatedness, appreciation of nature and non-human animals, and environment-friendly values (Studerus et al., 2011; Forstmann and Sagioglou, 2017; Nour et al., 2017; Watts et al., 2017; Kettner et al., 2019; Gandy et al., 2020; Pöllänen et al., 2022). These changes fit Schwartz et al. (2012) “universalism–nature” value category, defined as the preservation of the natural environment.

#### 4.1.5. Universal concern

Schwartz et al. (2012) value category “universalism–concern”—encompassing commitment to equality, justice, and protection for all people—is probably enhanced by the increased prosocial attitudes explored above. Moreover, psychedelic experiences often involve reflection on and imagination about moral and ethical issues and values. Watts et al. (2017, 534–535) report cases of moral deliberation in psilocybin experiences: one-third of 12 participants had insights into the ongoing refugee crisis, and some pondered climate change, and reported becoming concerned about global issues afterward. These processes of ethical reflection and imagination suggest reduced egocentric biases and a shift to more self-transcendent evaluative modes. As epitomized by one patient, “I got a wider perspective […] It helped me appreciate that the world is a big place, that there’s a lot more going on than just the minor things […] in my head” (Watts et al., 2017, 534). Masters and Houston (1966, 255) cite an early study in which one-third of the 194 participants reported increased interest in ethics 10 months after an LSD session. Shanon (2002) observes that ayahuasca intoxication tends to affect values and is often experienced as a lesson in morals:

Reflection about certain values and a sense of commitment towards them seems to be especially salient. Those reported by many individuals include personal responsibility, justice, and love. Also common is the appreciation of the significance of faith and hope, patience, and humility. Common is the appreciation that values—in particular, love and justice—are not confined to the province of human life but they also apply to existence at large and to the forces or beings that govern the universe. (p. 174)

Similarly, morally relevant themes can feature in psychedelic visions (Shanon, 2002, 173–175).

#### 4.1.6. Humility

In Schwartz et al. (2012), humility is a universal self-transcendent value related to recognizing one’s insignificance in the larger scheme of things. Notably, quiet ego and hypo-egoism are associated with the character trait of humility and putting the self in perspective (Leary and Guadagno, 2011; Wayment and Bauer, 2017), and Murdoch (1997, 99, 104) used humility as a prime example of non-egocentric virtue. Humility as both value and trait is integrally connected with reduced egocentricity and widened perspectives.

Shanon explicitly mentions humility as an important lesson of psychedelic experiences (2002, 159, 174). Many other features of psychedelic experiences—including awe (and associated “small self”), acceptance, gratitude, sense of connectedness and interconnectedness, spirituality, and relational embeddedness—are conceptually associated with humility (Shanon, 2002, 205; Hendricks et al., 2015; Belser et al., 2017, 16–17; Watts et al., 2017; Carhart-Harris et al., 2018b; Letheby, 2021, 197–204).

#### 4.1.7. Spirituality and sacredness

Letheby (2021, 197–204) argues that psychedelic experiences foster spirituality by enhancing the ability to recognize one’s place in the larger scheme of things, through developing broader perspectives and expanding attention beyond self-related concerns. Empirically, psychedelics are associated with an increased appreciation of spirituality (Lerner and Lyvers, 2006; Griffiths et al., 2018). A sense of sacredness and encounters with the sacred are common features of psychedelic experiences (Shanon, 2002, 156, 262; Griffiths et al., 2019; James et al., 2020). Furthermore, visionary and mystical experiences sometimes bring insights into values. These states are associated with *noesis*, a sense of gaining knowledge not mediated by reflective thought or sense perception. For example, Shanon (2002) reports an encounter with Supreme Good and a subsequent noetic insight into the unity of values, highly reminiscent of Platonic philosophy: “A major impression these visions had on me is the (Platonic) conclusion that ultimately, the ethical and the aesthetical as well as the true are the same” (p. 174). The similarity to Maslow’s B-values should also be noted. Mystical experiences, God encounter experiences, and similar psychedelic states can plausibly acquaintance one with deep intrinsic values of existence such as transcendent values postulated by many religious traditions (Pelser and Roberts, 2015; Griffiths et al., 2019), signaling a shift to self-transcendent modes of valuation.

### 4.2. Factors affecting values during the psychedelic experiences

#### 4.2.1. Self-transcendent experiences

Psychedelic experiences are a significant source of STEs (Yaden et al., 2017; Letheby, 2017, 2021; St. Arnaud, 2019; Isham et al., 2022). Psychedelics often induce various changes in the sense of self, ranging from experiences of connection to union experienced in mystical experiences (Millière, 2017; Millière et al., 2018; Letheby, 2021, 46–53; Nour and Carhart-Harris, 2017). The sensed boundary between self and the world might weaken, and one can experience empathy, affinity, blending, identification, and even unification with what is ordinarily other (Shanon, 2002, 205; Belser et al., 2017, 16–17; Yaden et al., 2017). Also commonly experienced are perceived interconnectedness; an increased sense of connectedness to oneself, nature, and the world; and states of *communitas* and social connectedness (Belser et al., 2017; Watts et al., 2017; Carhart-Harris et al., 2018a; Kettner et al., 2019; Blatchford et al., 2020; Forstmann et al., 2020; Kettner et al., 2021). Similarly, psychedelic experiences can bring enhanced meaningfulness of ordinarily uninteresting aspects of the world and enhanced present-centered attention, suggesting the release of salience from constraints of egocentricity (Huxley, 1954; Shanon, 2002, 61; Letheby, 2017; Letheby and Gerrans, 2017; Hartogsohn, 2018). Sense of awe, wonder, and the so-called overview effect are commonly reported expanded perspectives in psychedelic experiences (Shanon, 2002, 61–62; Yaden et al., 2016; Flanagan and Graham, 2017; Letheby, 2017, 637; Hendricks, 2018, 302–307).

#### 4.2.2. Role of STEs in value changes

Various STEs are associated with psychedelic value changes in all but two of the studies outlined in Table 2. In seven of the 15 studies, STEs were either statistically mediated or were statistically associated with observed changes in values. A connection is also suggested in the phenomenological reports of four studies and the discussions of findings in two studies. This evidence indicates that the self-transcendent perspectives and expanded self–other boundaries associated with STEs are likely to mediate long-term changes in values and life priorities. Congruently, STEs also mediate many other long-term benefits of psychedelics. In a meta-analysis of 15 therapeutic studies, Kałużna et al. (2022) found that experiences of unity and connectedness in psychedelic experiences strongly predicted long-lasting therapeutic outcomes. While ego-dissolution was a weaker predictor of therapeutic gains, many studies described it as a tool for gaining a wider perspective, reinforcing the hypothesis that perspectival widening results from reduced egocentric salience.

Mystical experiences have been observed to mediate changes toward increased prosociality and spirituality (Griffiths et al., 2008, 2018; Orlowski et al., 2022). Similarly, the literature suggests a connection between ego-dissolution and change toward more environment and animal-friendly values (Pöllänen et al., 2022). Forstmann and Sagioglou (2017, 1) suggest that people might become more environmentally friendly “by changing their self-construal in terms of an incorporation of the natural world.” In the longitudinal study aptly titled “From Egoism to Ecoism,” Kettner et al. (2019) found that ego-dissolutions mediated increases in nature-relatedness, suggesting a causal connection. If construed and experienced as less separate from nature, the self becomes embedded in the wider context of the natural world, and the intrinsic value of nature might be perceived as higher than under egocentric modes.

One prominent reason why mystical experiences and ego-dissolution might lead to changed values is that they bring an acute awareness of the transient nature of self, as this center of valuation temporarily ceases to exist. In an embodied and felt way, radical deconstruction of self might give *recognition of the nothingness* of ourselves—“the existential shock that attends the vivid apprehension of one’s own mortality” (Letheby, 2021, 187; see also Baillie, 2013, 187; Baillie, 2020). In mystical experiences and ego-dissolution, the sense of self and boundaries between oneself and the world can dissolve altogether, temporarily collapsing the ordinary frames of reference for egocentric pursuits (Swanson, 2016; Millière, 2017; James et al., 2020; Laukkonen and Slagter, 2021). Although everyone intellectually understands their mortality and embeddedness in a vast cosmos, psychedelic experiences can animate these facts by presenting them in a visceral, emotionally deep fashion (Grob, 2007, 213; Letheby, 2021, 184–191). Psychedelics can foster facing one’s mortality and radically resituating oneself as part of a wider whole with long-lasting effects (Gandy, 2017; Malone et al., 2018, 4; Schmid and Liechti, 2018; St. Arnaud, 2019; Sweeney et al., 2022).

Literature on awe suggests similar although lesser gestalt shifts in our perspective on self. Awe can situate the self as part of our wider world, causing a perspective of reduced self-importance termed “small self” in reaction to the vast stimuli transcending one’s current frames of reference, thus broadening our attention and perspective beyond egocentric and instrumental concerns (Vervaeke and Ferraro, 2013; Piff et al., 2015; Hendricks, 2018; Perlin and Li, 2020). Awe and the related perception of “small self” are likely to mediate many value changes, as these experiences allow for tuning into wider contexts of evaluation. Mulukom et al. (2020) observed that psychedelic-induced awe and sense of connection toward nature and humanity (but not the universe) led to increased affective empathy and reduced narcissistic tendencies. Moreover, St. Arnaud and Sharpe (2022) found that awe-proneness mediated positive adult development associated with psychedelics. Significantly, awe is also an important psychological mediator of psychedelics’ therapeutic effects (Hendricks et al., 2015).

#### 4.2.3. Increased motivational salience of values

As another possible mechanism of value change, psychedelic experiences might enhance the felt importance and motivating force (i.e., salience) of our values. Through enhanced meaning and meaningfulness and increased suggestibility, psychointegration effects, and visceral representation of knowledge, psychedelics can make our abstract convictions and conceptions experientially alive (Shanon, 2002, 242–255; Carhart-Harris et al., 2015; Hartogsohn, 2018; Letheby, 2021; Timmermann et al., 2022, 184–191). Transposing Letheby’s (2021), 188–190) notion of gaining a deeper knowledge of old facts, psychedelic experiences can acquaint us with our values in a deeper, more vivid, and embodied manner than is usually possible, enabling deeper and more meaningful experience of these convictions. Therefore, psychedelic experiences might better align our motivation and experiential salience with our values, perhaps leading to more committed action according to these values, as values gain more “incentive or motivational salience” (Ratcliffe and Broome, 2022, 51). Theoretically, activation of the 5HT2a serotonin system—the primary neurobiological mechanism activated by psychedelics—has been linked to active coping and enhanced capacity for change (Carhart-Harris and Nutt, 2017; Brouwer and Carhart-Harris, 2021), and might mediate the increased motivational salience of values and increases in value-laden striving.

This supposed increase of “volume” in value–salience connections might significantly contribute to (re)connecting to self-transcendent values and play role in other kinds of value changes as well, as it is conceptually independent of self-transcendence (see Section 4.4). Increases in the motivational salience of values are morally significant, as values should mandate us to experience certain things as strongly salient *and* solicit a response (*cf.* Siegel, 2014; Cavedon-Taylor, 2022, 15). It is morally problematic if a person’s values do not inform an embodied, experiential sense of salience and meaningfulness, nor motivate action (although not all values can be “active” at every moment; see Oyserman, 2015).

### 4.3. Connections between values and other long-term changes

Psychedelics can bring other significant long-term changes, such as increases in the traits of self-transcendence, mindfulness capacities, psychological flexibility, openness to experience, and alterations in neural networks. Together these findings support the notion that changes in values are associated with self-transcendence and reduced egocentricity and offer further theoretical possibilities to account for value changes.

#### 4.3.1. Trait self-transcendence

As discussed in Section 3.7, developmental self-transcendence is associated with self-transcendent values. Psychedelic use is associated with increased trait-level self-transcendence and correlated neural changes (Bouso et al., 2012, 2015; Révész et al., 2021). St. Arnaud (2019) explores the idea that the psychedelic mystical experiences might help to treat existential anxiety by fostering traits of self-transcendence. Expanded self–other boundaries are theoretically central to many notions of developmental self-transcendence, supporting the hypothesis that psychedelic changes in sense of self could lead to similar long-term changes (St. Arnaud, 2019). Significantly, St. Arnaud and Sharpe (2022) found an association between psychedelic use and positive adult development (a construct similar to developmental self-transcendence). Psychedelics further enhance many tendencies associated with the trait of self-transcendence, such as a sense of connection, gratitude, death transcendence, and meaning in life (Belser et al., 2017, 16–17; Watts et al., 2017; Griffiths et al., 2018; Schmid and Liechti, 2018).

#### 4.3.2. Mindfulness capacities

Both short-and long-lasting increases in mindfulness capacities support the idea that psychedelic experiences can induce changes in attentional capacities (Bouso et al., 2012; Soler et al., 2016; Radakovic et al., 2022). Smigielski et al. (2019a,b) conducted a double-blind intervention combining psychedelics and mindfulness training; they found that neurobiological changes in areas associated with self-relevant processing [such as the default mode network (DMN) and posterior cingulate cortex (PCC)] and ego-dissolution during psychedelic experiences mediated increases in mindfulness capacities for up to 4 months after the intervention. Sampedro et al. (2017) found that similar structural changes in the brain correlated with enhanced mindfulness capacities 2 months after ayahuasca intake. As mindfulness capacities involve decentered and present-moment-oriented modes of attention, these empirical findings strongly support the hypothesis that psychedelics can lead to long-lasting unselfing (i.e., reduced egocentric attention and salience). The development of mindfulness capacities is also empirically associated with both value-oriented life (Franquesa et al., 2017) and self-transcendence (Vago and David, 2012).

#### 4.3.3. Psychological flexibility

Psychological flexibility processes are one avenue to account for (re)connection to values and possibly other value changes. Psychedelics have been observed to increase psychological flexibility, a central construct of acceptance and commitment therapy (ACT), which involves a) connection and commitment to values and value-driven action, and b) a decentered sense of self (Davis et al., 2020). Studies exploring psychedelics’ impact on psychological flexibility processes suggest that psychedelics might help to shift flexibility-fostering modes of selfhood (i.e., perspective-taking self or self-as-context in ACT terminology; Hayes et al., 2019; Watts and Luoma, 2020; Whitfield, 2021). Converging with the framework presented here, ACT posits that these flexible modes of selfhood imply reduced identification with our self-conceptions and personhood (self-as-content) and foster living according to one’s values.

#### 4.3.4. Openness to experience

Some value changes might be explained by changes in personality factors, such as openness to experience. Multiple studies observe that the trait of openness to experience is increased by psychedelics (MacLean et al., 2011; Carhart-Harris et al., 2016; Nour et al., 2017; Erritzoe et al., 2018, 2019). Aspects of openness to experience might be related to changes in values. For instance, appreciation of art and nature is plausibly connected to “openness to aesthetics” (partly explained by reduced latent inhibition—the tendency to filter habituated sensory content from perception; St. Arnaud, 2021, 99). Openness to experience is also related to absorption, a tendency to pay total attention (McCrae, 2004), consonant with the unselfed mode of attention. Erritzoe et al. (2019) found that psychedelics have an especially strong effect on “openness to actions” and “openness to values,” the latter encompassing tolerance toward other people’s lifestyles and willingness to redefine one’s own values. These findings support both the notion of a general tendency for value change and specific change toward the universalist value of tolerance (i.e., acceptance and understanding of those different from oneself; Schwartz et al., 2012).

#### 4.3.5. Neurocognitive changes

Theoretically, many of the changes associated with unselfing can be explained by psychedelics’ ability to deconstruct the self-model—our ordinary phenomenal experience of being a separate self and the underlying neural and cognitive processes (Letheby, 2021). It is theorized that psychedelics weaken the power of models built by prior experience and that the self is a high-level prior central to our overall modeling of the world (Swanson, 2016, 2018; Letheby and Gerrans, 2017; Carhart-Harris and Friston, 2019). Huxley (1954) framed the ego as a “reducing valve” that filters our perception of the world into a utilitarian form useful for instrumental pursuits and proposed that psychedelics might temporarily relax this valve. His ideas have since been corroborated by neuroscientific research. In predictive processing terminology, self-related priors constrict evaluative processes and values; therefore, temporary radical destabilization and subsequent relaxation of (overweighted) self-related priors might do much of the work associated with self-transcendent value changes. Proposed outcomes of destabilizing high-level priors include increased context sensitivity and a more fluid ability to tune into various perspectives (Carhart-Harris et al., 2018b; Carhart-Harris and Friston, 2019).

From a neurodynamic viewpoint, reduction in egocentric attributions of salience and evaluations likely occurs through altered brain areas and networks associated with selfhood, such as the default mode network, posterior cingulate cortex, and salience network; changes in these correlates acutely and in the long-term with psychedelic STEs (Bouso et al., 2015; Letheby and Gerrans, 2017; Millière et al., 2018; Smigielski et al., 2019a). Similar neural changes are observed in meditation practices (Vago and David, 2012; Brewer et al., 2013; Letheby, 2022).

### 4.4. The role of contextual factors

#### 4.4.1. Psychedelic pluripotency

It is clear from various anecdotal accounts that there are mechanisms of psychedelic value change other than self-transcendence, and that self-transcendent values are not the only possible outcome. Psychedelics have been used by cults such as the Manson Family and Aum Shinrikyo, by neo-Nazis and the right-wing intelligentsia, and among bellicose Amazonian and Mesoamerican societies, with no apparent challenge to respective values or causing further enculturation towards these worldviews and values (Rios, 1996; Piper, 2015; Pace and Devenot, 2021, 6).^5^ These counter-examples disprove the claim that psychedelics inevitably lead to the unidirectional change of values.

To account for this “pluripotency” of psychedelic transformations, many explanations cite contextual factors—the immediate context of and the cultural matrix surrounding the use of psychedelics (Dupuis, 2021; Pace and Devenot, 2021). Psychedelics increase suggestibility and psychedelic experiences are highly sensitive to influences from contextual factors (Carhart-Harris et al., 2015; Hartogsohn, 2017, 2018, 2020; Carhart-Harris et al., 2018b; Eisner, 1997). Dupuis and Veissiere (2022) and Dupuis (2021) suggest that psychedelics might act as “super-placebos” and as “tools for cultural transmission” of beliefs and values, utilizing temporary cognitive openings created during experiences (see also Katz, 1978; Pace and Devenot, 2021, 6; Dupuis, 2022a). Thus, psychedelics could make one to adapt or emphasize various values, beliefs and political orientations provided by the social and cultural context of psychedelic use.

No existing research has conclusively determined the relative strength of directionality toward self-transcendent values and the amplification of specific cultural values (Langlitz et al., 2021). In general, to what extent does the observed directionality in psychedelic belief (Griffiths et al., 2019; Timmermann et al., 2021; Nayak and Griffiths, 2022) and value change (see Table 2) stem from social and cultural factors, personal background and intrinsic features of psychedelic experiences is an open question requiring further exploration.

Still, it is doubtful that a purely contextual or cultural account can explain all the observed patterns in the change of values. First, changes contravening (sub)cultural values are also possible (Wallace, 1956; Roseman and Karkabi, 2021). Second, if psychedelics reliably deconstruct the self-model, deconstruction of egocentric valuation modes and values and movement toward self-transcendent values is plausibly one higher-order constant across value changes occurring in different social and cultural settings.

Importantly, even if changes toward self-transcendent values are a higher-order constant of value change, they are still plausibly mediated by one’s worldview and epistemology and negotiated with one’s other values. Culturally mediated conceptions, beliefs, and models of the world affect how we flesh out what abstract values and good mean and what values imply for action (Murdoch, 1992, 323–325). For example, seen through the biasing lens of a particular ideology and worldview, even activities such as pursuing societal collapse or sacrificing humans to prevent apocalypse might be conceived as non-egocentric (on Aztecs and mushrooms, see Rios, 1996; on accelerationism, see Pace and Devenot, 2021); similarly, nature relatedness could be channeled to ecofascism, and allocentric widening to loyalty to a xenophobic ingroup (Pace and Devenot, 2021).

#### 4.4.2. Optimal conditions for value change

The argument presented here has clarified ways to understand cases where psychedelic STEs translate to long-term changes in values. As discussed in Section 3.9, the translation of momentary experiences into lasting values is far from automatic, many contextual factors likely mediate the relation between momentary experiences and long-term changes, and might hinder or amplify the change toward self-transcendent values.

Notably, St. Arnaud and Sharpe (2022) found that prior intentions and integration or post-experience reflection mediated whether psychedelic experiences led to positive adult development. Lasting value change is highly likely to be mediated by similar factors, as well as by the individual’s prior developmental stage (St. Arnaud, 2019). Relatedly, the importance of a “rich context” for moral neuroenhancement is stressed by Earp et al. (2017) and Earp (2018). Value changes might be most likely where psychedelic use is embedded in rich cultural contexts with a mature understanding of the good life and self-transcendence, and with ecologies of practice that can support a positive long-term change of values.

To maximize the wise and beneficial use of psychedelics, the contextual factors supporting self-transcendent value changes should be more closely explored. For example, the synergistic effects of psychedelic and meditative practices should be further investigated (Griffiths et al., 2018; Smigielski et al., 2019a,b; Heuschkel and Kuypers, 2020; Payne et al., 2021; Simonsson and Goldberg, 2022).

## 5. Discussion

### 5.1. Implications of the framework

To conclude, one plausible psychological or personal-level mechanism for psychedelic value change is better access to non-egocentric intrinsic and self-transcendent values via reduced egocentric biases in attention and salience. Both long-term changes in values and momentary STEs and perspectival shifts in psychedelic experiences can be plausibly explained through the same unselfing mechanisms. The idea that many value changes are toward self-transcendent values, resulting from processes and experiences of self-transcendence, is a parsimonious way to explain various value changes in a unified fashion, given that STEs are already a central proposed mechanism for psychedelics (Liester and Prickett, 2012; Letheby, 2021, 197, 202; Letheby, 2022; van Elk and Yaden, 2022) and self-transcendence in general (Maslow, 1971; St. Arnaud, 2019). Furthermore, this article’s theoretical integration supports the claim that temporary reductions in self-saliency and selfless experiences might sometimes translate into long-term self-transcendent changes in values and traits (*cf.* Millière et al., 2018; St. Arnaud and Sharpe, 2022). Although STEs do not automatically bring long-term changes in values and behavior, as many contextual factors mediate such changes.

Unselfing and changes toward self-transcendent values are existentially and morally significant phenomena. The proposed framework explains self-transcendent value changes as part of wider self-transcendent changes, seen by many theories as a desirable part of optimal human development (cf. St. Arnaud, 2019). Self-transcendent values are important or even central for good life and morality (if one does not subscribe to extreme forms of moral egoism or nihilism). Similarly, states of self-transcendence and reduced egocentricity have been empirically linked to increased wellbeing (Leary and Guadagno, 2011, 139; Wong, 2016; Yaden et al., 2017; St. Arnaud, 2019; Isham et al., 2022).

Psychedelics and other techniques for achieving self-transcendence might thus be important for living good lives, especially where a person lacks access to non-egocentric perspectives. It is notoriously difficult to rationally argue why certain intrinsic values (such as nature or art) should be adopted for non-instrumental reasons; these values must be personally and experientially recognized and understood. Psychedelics might be especially helpful for generating such axiological insights and expanded salience perspectives.^6^

My argument supports the interpretation that psychedelic experiences sometimes expand one’s view into fundamental values of our lives. With the relaxation of egocentric attributions of salience, evaluative context may widen enabling one to better grasp self-transcendent values and to connect to one’s core values. Visiting psychedelic states might allow for existentially and morally important direction-giving glimpses and the activation of self-transcendent values, even if more is needed for inner transformation. Thus, psychedelics might sometimes provide a potent source of proleptic rationality, giving reasons to aspire toward valuable directions in life (Callard, 2018; Letheby, 2021; 198). Psychedelic experiences might enhance moral agency by reducing overt attribution of egocentric salience impeding one’s moral agency, by increasing epistemic access to non-egocentric values and by aligning one’s salience and motivation with them. In this way psychedelic experiences might straighten egocentric misconceptions and biases about the good.

#### 5.1.1. Justification of value changes

The proposed framework provides *prima facie* philosophical justification for psychedelic value changes toward self-transcendent values. According to this framework, self-transcendent value changes occur through personal-level processes that are epistemically and normatively justified, and respect individual autonomy, as enhanced access to self-transcendent perspectives and values gives reasons for value changes (*cf.* Earp et al., 2017; Lavazza, 2017; Langlitz et al., 2021). However, the matter of psychedelic pluripotency warrants some concern. If value changes sometimes stem from being exposed to values and value-laden cultural beliefs and worldviews from the sociocultural setting of psychedelic use, the ethical issues whether psychedelic value changes respect autonomy and are desirable become more complex. It is possible that some contexts of use could lead to coercive or semi-coercive value changes that do not respect individual autonomy (*cf.* Earp et al., 2017; Lavazza, 2017; Dupuis, 2021, 2022a,b; Dupuis and Veissiere, 2022). Thus, how social and cultural influences affect psychedelic value changes should be further explored.

The risks associated with the possibility of suggestive influences involved in the change of values highlight the need for epistemic responsibility, enhanced informed consent, and further inquiry into the wider epistemic and moral context for psychedelic use, as Smith and Sisti (2021), Langlitz et al. (2021), and Letheby (2021) have already advocated. Moreover, Timmermann et al. (2022) proposed psychedelic apprenticeship, entailing practices of intersubjective guidance, validation, and inquiry into the sometimes-controversial knowledge gained during psychedelic. This is also highly relevant for value changes. Psychedelics should be optimally embedded in wise cultural, epistemic, and therapeutic contexts giving room for moral reflection and inquiry into possible value changes.

#### 5.1.2. Societal implications and moral neuroenhancement

Given the challenges of climate change and other existential questions facing humanity, the potential of psychedelics to enhance self-transcendence and self-transcendent values should be further explored from a societal viewpoint. For example, increased nature-relatedness is among the strongest predictors of environment-friendly behavior (Kettner et al., 2019). The possibility of self-transcendent value changes is a prominent reason why psychedelics might be useful as a pharmacological means to enhance humans’ moral state (Earp et al., 2017; Earp, 2018). The proposed framework could unify and enrich many proposals in the psychedelic moral enhancement literature (Tennison, 2012; Ahlskog, 2017; Earp, 2018, 18–19; Ballesteros, 2019; Germann, 2019; Lange and Marie, 2021; Kirkham and Letheby, 2022).

### 5.2. Future directions and limitations

#### 5.2.1. Toward a theory of psychedelic value change

To build a comprehensive theory of psychedelic value change, future work must identify the various types of value changes and mechanisms and explore their interactions. For example, increased suggestibility (Carhart-Harris et al., 2015), the influence of sociocultural factors (Hartogsohn, 2020; Dupuis, 2021; Dupuis, 2022b), alterations in meaning processing, and fluidity of cognition (Shanon, 2002, 143, 243, 340; Hartogsohn, 2018; Vervaeke et al., 2018) might all play a role irrespective of unselfing. Similarly, reduced discrepancy between one’s values and salience, increased positive affect and vitality, personality factors such as openness and optimism, and changes in emotional processing and motivation might also play a part (Winkelman, 2001, 2017; MacLean et al., 2011; Carhart-Harris et al., 2016; Vollenweider and Smallridge, 2022). There are likely other mechanisms to be discovered.

This article focused on the possible psychological mechanisms of value change associated with unselfing. An in-depth exploration of how the psychological or experiential levels of value change fit with other levels of explanation (e.g., neuroscientific) is a worthwhile future research task. The possible mechanisms on psychological, socio-cultural, and neurobiological levels, and the relations between various levels of explanation, should be explored (*cf.* van Elk and Yaden, 2022). Integrating an existing neuroscientific understanding of values (e.g., Moll et al., 2015) with theoretical and neuroscientific models of psychedelics, such as the models based on the predictive processing paradigm (Letheby and Gerrans, 2017; Carhart-Harris and Friston, 2019), could yield many insights.

Another important theoretical question is whether psychedelic value changes fit value dimensions or polarities other than those explored here (i.e., self-enhancement versus self-transcendence). For example, psychedelic value changes in political attitudes and openness to values might map to the dimension “openness to change” versus “conservation” in Schwartz et al.’s (2012) value theory (Nour et al., 2017; Lyons and Carhart-Harris, 2018; Erritzoe et al., 2019).

#### 5.2.2. Empirical inquiry into value change

Another important research avenue is to operationalize and empirically test the hypotheses presented in this article. It is especially important to explore the connections among various psychological constructs associated with unselfing and value change. Direct psychometric measurement of various constructs related to unselfing (such as quiet ego and ego development) and measurements of value changes (for instance, using Schwartz’s scales and ACT value subscales) would provide further evidence for the questions posed herein.

#### 5.2.3. Philosophical issues

Similarly, there are many pressing philosophical questions concerning value change. Future research should investigate the justification of psychedelic value changes, their relevance for moral enhancement literature, and the normative and metaethical questions raised (such as which conceptions of values and of the good life are congruent with the proposed framework).

#### 5.2.4. Limitations

This article is based on a broad-stroke review of multiple bodies of literature, suggesting hypotheses on the psychological mechanisms of psychedelic value change. To test these ideas against solid evidence, a more rigorous empirical inquiry into psychedelic value changes is required. As this article is based on a selective reading of philosophy and broad bodies of empirical and theoretical literature, efforts to explore alternative theoretical and philosophical hypotheses to explain the findings of various studies should be conducted. It should also be noted that most prior studies into psychedelic value changes are either correlational or relatively small scale and that there are many open empirical questions concerning the change of values patterns (for example, regarding the relative influence of cultural context; see Langlitz et al., 2021). Moreover, many studies suffer from self-selection bias and draw individual participants from relatively narrow socio-economic backgrounds, who share a cultural worldview and hold a certain set of values.

## 6. Conclusion

This article establishes a plausible connection between psychedelic experiences and value changes toward self-transcendent values. According to the proposed framework, these value changes stem from unselfing—a reduction in egocentric attributions of salience, enabling (re)connection to self-transcendent values. I argue that this increases our capacity to pay attention to reality outside the self and can widen our evaluative context. The central idea is that self-transcendent values are inherently tied to the goods of these various self-transcendent evaluative contexts. Thus, by opening to these wider contexts, an individual gains enhanced epistemic access to self-transcendent values.

The framework fits with the reviewed insights from statistical, theoretical, and qualitative research on psychedelic value changes. Psychedelics can enhance reconnection to values, esthetic values, benevolence/prosocial values, universalism values associated with the good of mankind and the natural world, humility, and spirituality. Empirical and theoretical accounts of psychedelics support the connection between these self-transcendent changes and various STEs (such as awe and mystical experiences), alterations in self-construal, and other psychological and neural changes typically induced by psychedelics. Furthermore, independently of psychedelic research, STEs are linked to reduced trait-level egocentricity and self-transcendent values. Convergence between various theoretical constructs suggests that morally and existentially relevant long-term changes can occur through reducing egocentricity and that STEs can contribute to these processes. If the proposed framework is correct, psychedelic value changes have potential ethical significance and are justified, although these philosophical issues warrant further investigation.

Although the presented evidence indicates robust theoretical and empirical associations between reduced egocentricity and change in values, there are many cases where STEs do not lead to value change. Thus, the personal and contextual factors mediating the link between experiences and long-term value changes need further exploration. Psychedelic value change is supposedly optimal in well-planned, rich moral contexts and in combination with other supporting practices. Future research should empirically explore the hypotheses presented in this article and chart the relation between self-transcendence and other possible mechanisms of value change.

## Data availability statement

The original contributions presented in the study are included in the article/supplementary material, further inquiries can be directed to the corresponding author.

## Author contributions

The author confirms being the sole contributor of this work and has approved it for publication.

## Funding

Signe & Ane Gyllenberg Foundation funded writing this article in fall 2022 by funding the author’s doctoral research (grant number 5848). University of Helsinki funded the open access publication fees.

## Conflict of interest

The author declares that the research was conducted in the absence of any commercial or financial relationships that could be construed as a potential conflict of interest.

## Publisher’s note

All claims expressed in this article are solely those of the authors and do not necessarily represent those of their affiliated organizations, or those of the publisher, the editors and the reviewers. Any product that may be evaluated in this article, or claim that may be made by its manufacturer, is not guaranteed or endorsed by the publisher.

## Footnotes

^1^Schwartz et al.’s (2012) value theory is an empirically based theory of culturally universal values. According to its various formulations, culturally universal values are grouped into either 10 or 19 core values forming a circumplex structure, where values are in orthogonal relation or inversely correlated to each other (i.e., scoring high on "A" values implies scoring low on "B" values). Self-transcendent and self-enhancement values are orthogonal categories in Schwartz’s theory.

^2^https://www.opendemocracy.net/en/oureconomy/psychedelics-systems-change-could-drugs-help-us-save-planet/ (Accessed 16.3.2023).

^3^Egocentricity and selflessness can have various connotations (Millière, 2020). Egocentricity is often contrasted with the capacity for taking on others’ perspectives (e.g., Bukowski et al., 2022). The egocentric attributions of salience do not concern solely the capacity for perspective-taking. A person with high perspective-taking ability might still be egocentric, for example, when narcissistically taking another’s perspective to manipulate them. Sometimes egocentricity is used to describe certain perceptual modes, as distinction between egocentric versus allocentric modes of visual perception (Fiehler et al., 2014; Letheby and Gerrans, 2017, footnote 3). This phenomenon is related to salience attribution in perceptual processes. However, I use a broader meaning of egocentricity.

^4^Value realism is one possible metaethical stance to ground this article’s arguments. Values as human universals (i.e., existing not in the fabric of cosmos but in our shared make-up) and cultural relativism are also congruent metaethical positions. However, nihilism, subjectivism, error-theory, and similar stances that deflate or deny the existence of objective or intersubjective values are probably not coherent with present framework (Kähönen, 2020, 72–76, 96–106).

^5^See also Langlitz’s (2020) ‘Rightist Psychedelia’: https://culanth.org/fieldsights/rightist-psychedelia (Accessed 16.3.2023).

^6^Cf. Whiteley (2022) and her concept of "harmful salience perspectives." STEs might generate the opposite: existentially and morally uplifting salience perspectives.
